# Developmental and Lactational Exposure to Dieldrin Alters Mammary Tumorigenesis in Her2/neu Transgenic Mice

**DOI:** 10.1371/journal.pone.0004303

**Published:** 2009-01-28

**Authors:** Heather L. Cameron, Warren G. Foster

**Affiliations:** Department of Obstetrics and Gynecology, McMaster University, Hamilton, Ontario, Canada; Health Canada, Canada

## Abstract

Breast cancer is the most common cancer in Western women and while its precise etiology is unknown, environmental factors are thought to play a role. The organochlorine pesticide dieldrin is a persistent environmental toxicant thought to increase the risk of breast cancer and reduce survival in the human population. The objective of this study was to define the effect of developmental exposure to environmentally relevant concentrations of dieldrin, on mammary tumor development in the offspring. Sexually mature FVB-MMTV/neu female mice were treated with vehicle (corn oil), or dieldrin (0.45, 2.25, and 4.5 µg/g body weight) daily by gavage for 5 days prior to mating and then once weekly throughout gestation and lactation until weaning. Dieldrin concentrations were selected to produce serum levels representative of human background body burdens, occupational exposure, and overt toxicity. Treatment had no effect on litter size, birth weight or the number of pups surviving to weaning. The highest dose of dieldrin significantly increased the total tumor burden and the volume and number of tumors found in the thoracic mammary glands. Increased mRNA and protein expression of the neurotrophin BDNF and its receptor TrkB was increased in tumors from the offspring of dieldrin treated dams. This study indicates that developmental exposure to the environmental contaminant dieldrin causes increased tumor burden in genetically predisposed mice. Dieldrin exposure also altered the expression of BNDF and TrkB, novel modulators of cancer pathogenesis.

## Introduction

Breast cancer is the most prevalent cancer in women, and although the incidence varies with age, geography, ethnicity and socioeconomic status, the lifetime risk of developing breast cancer is 1 in 9, with 1 in 28 women expected to die from breast cancer [1], [2]. The geographic disparities in rates of breast cancer taken together with studies of twins and migrants have yielded compelling evidence that environmental factors play an important role in the pathobiology of breast cancer [3]–[5]. Indeed, it is thought that as much as 60% of breast cancer has an environmental etiology [4], [6], with strong toxicological evidence linking a number of ubiquitous environmental contaminants to breast cancer based on their ability to interact with or disrupt hormone signalling pathways or alter mammary gland development, thus altering susceptibility to mammary carcinogenesis [7], [8].

Of the environmental toxicants linked to breast cancer, dieldrin is of particular interest owing to evidence of human exposure and association with increased breast cancer rate and decreased survival in women with the highest exposures [9]–[11]. However the epidemiological literature is equivocal and mechanisms poorly defined. A large prospective study in a Danish population found a two-fold increased risk of developing breast cancer (adjusted odds ratio 2.05 [95% CI 1.17–3.57]) in women with the highest dieldrin exposure [10] with poorer overall survival and increased mortality in breast cancer patients (RR, 2.61 [95% CI 0.97–7.01]) [9]. While this positive association was not observed in several smaller studies [12], [13], the strong statistical correlation observed in the Danish studies taken together with results of animal studies demonstrating that dieldrin alters mammary gland development following developmental exposure [14], indicates the need to further investigate this association. Dieldrin is an organochlorine pesticide whose use was banned in North America over twenty years ago, but it is still widely detectable within the domestic and imported food supply [15]. Detectable levels of dieldrin are reported in over 90% of the human population in serum, adipose tissues and breast milk [16], [17] with an estimated mean daily intake of 0.5 µg/day [18], [19] leading to human burdens of 117–289 ppb [17], [20]. Thus while epidemiological evidence for an association between dieldrin and breast cancer is not conclusive, existing evidence does indicate a cause for concern and highlights the need to understand the potential role of dieldrin in breast cancer pathogenesis, particularly given the long natural history of breast cancer and ongoing worldwide exposure. Moreover, adequate study of toxicant exposures during critical windows of mammary gland development are necessary given the higher susceptibility to environmental toxicants in developing mammary structures and that dieldrin readily crosses the placental barrier and is excreted in breast milk leading to its accumulation in fetal and neonatal adipose tissues including the mammary glands [21]–[23].

The ability of dieldrin to act as a xenoestrogen has been proposed to underlie its ability to alter mammary carcinogenesis and associated mortality [10], [24], and is based largely on its ability to increase proliferation in estrogen receptor-α (ER-α) positive MCF-7 breast cancer cells [25]. However, dieldrin is a weak estrogen, with 10^−6^ the potency of estradiol making it likely that dieldrin acts via additional mechanisms [24]. Indeed dieldrin has been shown to activate alternative estrogen pathways mediated by ERβ and GPR30. We have previously demonstrated dieldrin increased attachment-independent survival in ER-α negative breast cancer cells [26] supporting the notion that dieldrin acts via pathways independent of traditional ER-α signaling.

We hypothesized that dieldrin modulates breast cancer pathogenesis by altering expression of novel growth factors thought to be involved in cancer progression. Specifically we hypothesized that expression of the neurotrophins and their cognate receptors is increased by dieldrin exposure. The neurotrophin growth factors and their receptors have recently been shown to play a role in mammary carcinomas as well as several human malignancies including pancreatic and pulmonary carcinoma [27]–[29]. The neurotrophins include nerve growth factor (NGF), brain-derived neurotrophic factor (BDNF), neurotrophin-3 (NT-3) and neurotrophin-4/5 (NT-4/5). While the neurotrophins share a common low affinity receptor (p75NTR), they also signal via three high affinity tyrosine kinase receptors (Trks); NGF preferentially activates TrkA, BDNF and NT-4/5 prefer TrkB and NT-3 preferentially binds TrkC. Although classically considered to be neuronal growth factors promoting neuronal survival and differentiation, the neurotrophins and their receptors are widely expressed in non-neuronal tissues [30]. Increased neurotrophin/neurotrophin receptor expression has recently been demonstrated in mammary carcinoma [26], [31]–[33], where they are thought to play a role in survival and proliferation of cancer cells [29], [34].

A major limitation of human studies is the inability to control for exposure, latency and confounders, therefore well designed animal studies are essential for understanding the relationship between exposure and altered cancer pathogenesis. However, most contemporary animal studies fail to account for critical windows of exposure, and relevant concentrations of test chemicals representative of human exposure. Moreover, we propose that testing on a background of risk may yield results of greater relevance to human health.

In this study we examined the effect of developmental and lactational exposure to dieldrin in a transgenic mouse model of spontaneous mammary tumor formation. In this model the *neu* oncogene, homologous to the human oncogene *ErbB2* (*Her2/neu*), under the control of the mouse mammary tumor virus long terminal repeat (MMTV) promoter which is active throughout mammary development, leads to the overexpression of constitutively active oncogenic *neu* [35]. The *c-neu* gene encodes Erb-B2, a trans-membrane glycoprotein (185 kDa) with tyrosine kinase activity closely related to epidermal growth factor receptor (EGFR) [36], and is overexpressed in twenty to thirty percent of primary human breast cancers [37]. In this model, mice develop spontaneous adenocarcinomas after a short latency period and are a good model for human breast cancer [35]. In this study we tested whether gestational and lactational exposure to environmentally relevant concentrations of dieldrin altered the formation of mammary tumors and whether the resulting mammary tumors showed altered expression of the neurotrophins and their receptors. The findings of this study support a role for dieldrin as a promoter of breast cancer pathogenesis and activation of the neurotrophin signalling pathway thought to be important in mammary cancer pathogenesis.

## Results

**Figure 1:**
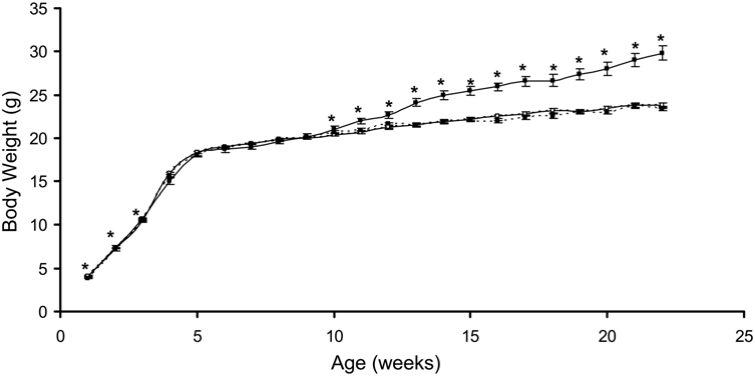
Mean body weight. Mean body weight (g) of mice exposed to either the vehicle (solid square dashed line) or dieldrin 0.45 µg/g BW (hollow square dashed line), dieldrin 2.25 µg/g BW (hollow square, solid line) and dieldrin 4.5 µg/g BW (solid square, solid line) throughout gestation and lactation. Although there we no differences in body weight between groups at birth, low dose dieldrin (0.45 µg/g BW) caused a modest increase in body weight from weeks 1 through 3 after which bodyweight did not differ from control. Dieldrin 4.5 µg/g BW increased body weight at 11 weeks continuing until sacrifice at 22 weeks. Data are presented as the mean±(SEM). Data analyzed by ANOVA, * *p*<0.05 compared to vehicle control.

Pregnancy rate was significantly reduced by the highest dose of dieldrin (4.5 µg/g BW) with fewer than 50% of females becoming pregnant in this treatment group with no effect observed in the lower dose treatment groups (Table S1). Despite this reduction in pregnancy rate, dieldrin treatment had no effect on the sex ratio or number of viable pups at delivery, nor on the number of male or female pups alive at postnatal day 7 or at weaning (Table S1). Furthermore, dieldrin showed no signs of general toxicity, as assessed by body weight, posture, coat, lacrimation and startle response. Body weight was affected by low dose dieldrin (0.45 µg/g) with a modest increase in postnatal body weight from weeks 1–3 after which body weight did not differ from control for the remaining 19 weeks (Figure 1). Mice treated with the highest dose of dieldrin demonstrated significantly increased body weights from week 11 and continuing until sacrifice at 22 weeks when mean body weight differed by over 6 g (Control 23.6±2.59 g compared to dieldrin 4.5, 29.8±3.77 g) (Figure 1). Liver and kidney weight measured at necropsy did not reveal any effect of dieldrin treatment on organ weight when calculated as a percentage of body weight (data not shown).

At sacrifice >90% of all mice had palpable mammary tumors, with tumors in the thoracic mammary glands being more prevalent than inguinal tumors (Table 1). The total tumor burden per mouse was significantly increased by dieldrin 4.5 µg/g treatment compared to control (Table 1) with treated mice burdened with 65% more tumors. The highest dose of dieldrin led to both an increased number of thoracic tumors per mouse and an increased volume of incident tumors. In contrast, the mean number of inguinal tumors was not significantly altered, and the resulting volume of incident tumors was significantly decreased in the inguinal compartment for the dieldrin 4.5 µg/g treated mice. No significant changes in tumor number or volume were observed in the dieldrin 0.45 and 2.25 µg/g treatment groups. Preliminary assessment of tumors arising in ovaries and liver assessed by histology revealed an increased incidence of ovarian tumors in dieldrin 2.25 µg/g (7.5%) and 4.5 µg/g (10.5%) versus control (2.6%). Increased liver tumors were also found in both higher dose dieldrin groups, with liver tumors occurring in 18.8% of dieldrin 2.25 µg/g treated mice and 52.6% of dieldrin 4.5 µg/g treated mice compared with 11.8% of control mice.

**Table 1:** Tumor number and volume of offspring at sacrifice (22 weeks).

	n	Total Number	Inguinal Tumors		Thoracic Tumors	
			Number	Volume	Number	Volume
Vehicle	84	4.62±0.60	0.75±0.13	15.61±6.33	3.81±0.51	49.12±16.09
Dieldrin 0.45 µg/g BW	79	4.82±0.43	1.00±0.15	8.62±1.84	4.24±0.41	45.55±11.82
Dieldrin 2.25 µg/g BW	81	4.54±0.43	0.73±0.12	12.41±5.54	3.82±0.36	18.28±5.85
Dieldrin 4.5 µg/g BW	19	7.58±1.24*	1.21±0.31	8.68±2.31*	6.37±1.06*	77.30±35.49*

Data expressed as Mean±SEM and differences between treatment groups were determined by one way ANOVA and appropriate post hoc test. Differences with a *p*<0.05 are indicated by a ^*^. n = number of mice/group, tumor volume expressed in mm^3^.

**Figure 2:**
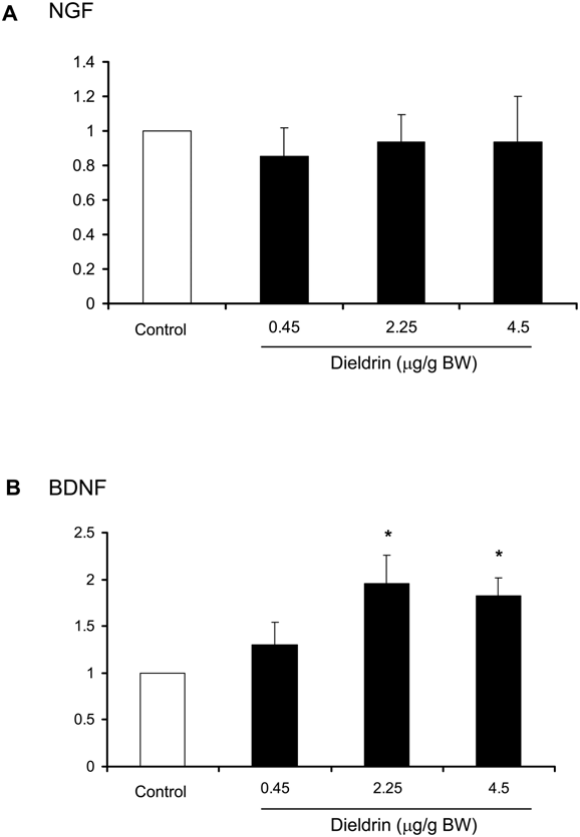
Dieldrin increases expression of BDNF mRNA. Mammary tumors from mice treated with vehicle or dieldrin (0.45, 2.25 and 4.5 µg/g BW) throughout gestation and lactation were processed for RNA extraction and cDNA prepared by reverse transcription. Induction of neurotrophin mRNA (NGF and BDNF) was determined by real time quantitative PCR. Expression of GAPDH was used for normalization. Each data point is the mean of at least 6 specimens±SEM. Data analyzed by ANOVA, * *p*<0.05 compared to vehicle control.

**Figure 3:**
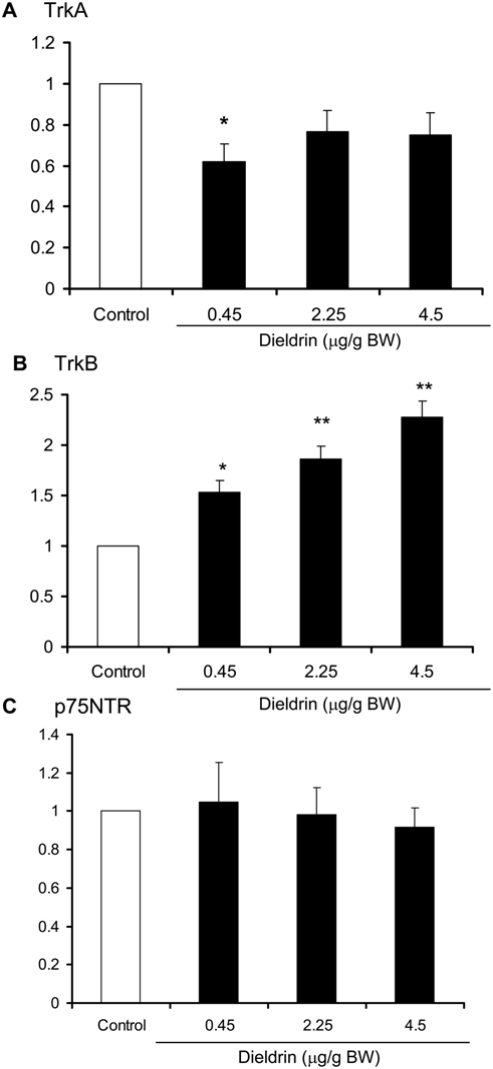
Neurotrophin and neurotrophin receptor mRNA expression in mammary tumors. Dieldrin increases expression of TrkB mRNA. Mammary tumors from mice treated with vehicle or dieldrin (0.45, 2.25 and 4.5 µg/g BW) throughout gestation and lactation were processed for RNA extraction and cDNA prepared by reverse transcription. Induction of neurotrophin receptor mRNA (TrkA, TrkB and p75NTR) was determined by real time quantitative PCR. Expression of GAPDH was used for normalization. Each data point is the mean of at least 6 specimens±SEM. Data analyzed by ANOVA, * *p*<0.05, **p<0.01 compared to vehicle control.

**Figure 4:**
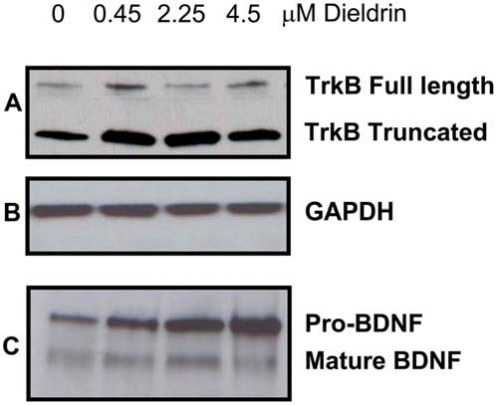
TrkB and BDNF protein expression in mammary tumors. Dieldrin treatment increases TrkB and BDNF protein expression in resulting mammary tumors measured by Western immunoblot. Tumors were sonicated on ice followed by protein extraction, electrophoresis and immunoprobing for TrkB, BDNF and GAPDH. Blots representative of at least four independent experiments.

Dieldrin treatment altered the expression profile of the neurotrophins and the neurotrophin receptors in mammary tumors. Based on quantitative RT-PCR, mRNA expression of the TrkA ligand NGF was unaffected by dieldrin treatment (Figure 2). In contrast, the TkrB ligand, BDNF was significantly increased by 2.25 and 4.5 µg/g dieldrin treatment (Figure 2). Expression of TrkA mRNA was reduced by dieldrin; a significant reduction was observed in the lowest dose group while a trend towards decreased expression was observed in the other dieldrin treatment groups (Figure 3). In contrast, TrkB mRNA was significantly increased in a dose-dependent manner in all three dieldrin treatment groups (Figure 3). Expression of the low affinity receptor p75NTR was unaffected by dieldrin treatment (Figure 3). Increased TrkB and BDNF protein expression in tumors from dieldrin treated animals was confirmed by Western blot (Figure 4).

**Figure 5:**
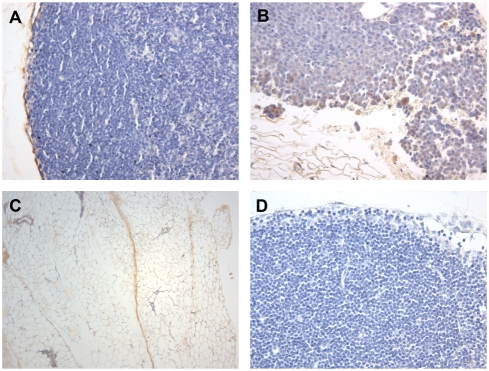
TrkB expression in mammary glands and tumors. TrkB protein expression in mammary glands and tumors. Immunohistochemical staining of formalin fixed mammary glands containing tumors demonstrates TrkB protein expression in both epithelial and stromal compartments. (A) TrkB positive myoepithelial cells encapsulating a TrkB-negative tumor (20×). (B) TrkB positive tumors demonstrating positivity at the outer edges of the tumor and positive adjacent stroma (20×). (C) TrkB positive mammary gland innervation (4×). (D) Negative control (20×).

To determine the tissue expression pattern of TrkB protein within mammary tumors and the mammary gland, immunohistochemistry was performed on thoracic mammary glands containing tumors (Figure 5). Figure 5A demonstrates the solid nature of a typical epithelial adenocarcinoma-like tumors in the mammary gland, characterized by prominent nuclei with little cytoplasm. Within the normal structures of the mammary gland, TrkB protein was present in myoepithelial cells adjacent to the tumors as well as in the surrounding stroma (Figure 5A). Mammary tumors expressing TrkB demonstrated a distinct pattern of positive staining where cells on the outermost edges of the tumor were more intensely stained than the interior of the tumor (Figure 5B). TrkB-positive stroma adjacent to the TrkB positive tumors was also noted (Figure 5B) as well as TrkB-positive gland innervation (Figure 5C).

## Discussion

Although dieldrin has been hypothesized to contribute to breast cancer incidence and associated mortality, the mechanisms for this observation have not been adequately addressed, nor has the contribution of developmental exposure been examined. The present study was designed to examine the ability of developmental and lactational exposure to dieldrin to alter mammary tumorigenesis in a model of spontaneous mammary tumor formation. This study demonstrates that dieldrin increases the formation of mammary tumors and alters the expression profile of neurotrophins/neurotrophin receptors in resulting tumors. While a role for dieldrin in tumor initiation remains to be established, this study supports the hypothesis that dieldrin exposure during critical windows of development against a background of risk increases mammary tumor development.

Dieldrin administration did not result in any signs of general toxicity in the dams or offspring, although the pregnancy rate in the high dose cohort was significantly reduced with only half of the females achieving pregnancy. In mice, dieldrin was previously found to reduce pregnancy rates although smaller litter size was a more common consequence of exposure [38]. In contrast, we did not observe any alterations in litter size, sex ratio or pup survival. Although pups did not differ in birth weight, the high dose dieldrin group demonstrated an increase in body weight at 11 weeks that continued until sacrifice at 22 weeks. This increase in body weight led to a final mean difference of 6 g/animal (compared to control) at sacrifice. We believe that this increased body weight is a consequence of the increased volume (and associated mass) of the mammary tumors occurring in this group. Although the two lower dieldrin dose groups did not alter total tumor development, the high dose treatment resulted in an increased overall burden of tumors per animal (7.58±1.24 vs. 4.62±0.60). In all treatment groups, tumors were more prevalent in the thoracic mammary glands than those in the inguinal region and were also much larger in this compartment. High dose dieldrin treatment resulted in significantly more thoracic tumors per animal that were also significantly larger compared to control, while the other dieldrin treatment groups did not significantly alter incidence or size of tumors. Interestingly, high dose dieldrin led to a decrease in inguinal tumor volumes, although no reduction in tumor incidence was observed. The size of the mammary fat pad at the inguinal versus thoracic site may also explain this discrepancy, with more room to accommodate tumor growth in the thoracic compartment. As such, we do not interpret this finding to be a protective effect since there was an increase in overall tumor burden and no reduction in incidence at the inguinal site. Furthermore, treatment with the two higher doses of dieldrin resulted in a dramatic increase in ovarian and liver tumors, with nearly a five fold increase in tumors in the dieldrin 4.5 treatment group compared to control. These data reveal that dieldrin treatment not only increases mammary tumorigenesis but tumorigenesis in ovary and liver, both estrogen-responsive organs. Ongoing studies aim to further elucidate the cause(s) of these extra-mammary tumors.

The role of novel growth factors in dieldrin-induced mammary tumor promotion was investigated in this study, and focused on expression changes in the neurotrophin/neurotrophin receptor family. In the present study all resulting mammary tumors expressed mRNA for the neurotrophins NGF and BDNF and neurotrophin receptors TrkA, TrkB and p75^NTR^. The neurotrophins and neurotrophin receptors have recently been implicated as novel mediators of carcinogenesis in many tissues. A role for the neurotrophins and their receptors in mammary carcinogenesis has previously been examined, and although the data are inconsistent, they do support a role for altered expression of these factors in cancer progression [33], [34]. Furthermore, we demonstrate that the tumors occurring in the dieldrin treated animals have an altered neurotrophin/receptor profile. More specifically, tumors occurring in animals in the high dose dieldrin group show significantly increased levels of BDNF and TrkB mRNA suggesting increased signaling via this pathway. Immunohistochemical staining demonstrated TrkB protein expression in tumor epithelial cells at the outer edges of tumors, as well as adjacent stroma and glandular epithelium. Western immunoblot confirmed the increased mRNA expression translated to increased protein expression in mammary tumors with both TrkB and BDNF expression increased in mammary tumors from dieldrin treated mice. Increased expression of TrkB/BDNF may indicate increased neo-neurogenesis, wherein tumors initiate their own innervation by the release of neurotrophic factors [39]. Although this hypothesis was not directly investigated in this study, our results support increased tumor expression of at least one neurotrophin. Increased TrkB protein expression was recently observed in breast cancer specimens compared with normal breast tissue [26], [33], and although a correlation with clinical outcomes was not assessed in these studies, elevated TrkB expression in neuroblastoma is correlated with a chemo-resistant phenotype [40], with increased mortality in Wilm's tumor [41] and is a predictor of distant metastases and prognosis in gastric cancer [42]. Signaling via the BDNF/TrkB pathway stimulates pro-survival signals, resistance to anoikis and altered cellular aggregation, all features of cancer cells and prerequisites of metastases formation [43]. While the role of TrkB/BDNF signaling in breast cancer is currently unknown, evidence from other malignancies supports a role for this pathway in tumor progression and clinical prognosis and thus merits further exploration. Increased angiogenesis, invasiveness and suppression of anoikis resulting from BDNF/TrkB signaling contributes to the aggressive phenotype of neuroblastoma tumors and is a likely mechanism for altered mammary tumorigenesis. The direct or indirect mechanisms by which dieldrin treatment in our model leads to increased expression of these factors in resulting mammary tumors is currently unknown and under investigation. In general, it is also currently unknown what factors regulate neurotrophin and neurotrophin receptor expression in normal and malignant breast tissues.

Dieldrin treatment did not alter expression of p75^NTR^ mRNA in mammary tumors although TrkA expression was decreased. Previous studies examining p75^NTR^ and TrkA expression in breast tissue and carcinoma have been conflicting, correlating increased expression to both good and poor prognosis [44]–[46]. Our study implicates TrkB signaling in mammary carcinogenesis which has not yet been adequately investigated particularly given its importance in cell survival and resistance to anoikis.

The mechanisms mediating dieldrin-induced tumor promotion in mammary carcinoma are currently unknown although several consequences of dieldrin exposure are themselves implicated in tumor promotion including activation of estrogen signaling pathways, oxidative stress [47], and inhibition of intercellular communication [48]. Activation of estrogen signalling pathways, specifically via ERα has been the preferred hypothesis since dieldrin is known to stimulate classical nuclear ERα-mediated signaling, although weakly. Alternative estrogen-dependent pathways mediated by ERβ or GPR30 are potential candidate pathways since estrogen is known to interact with these receptors and are currently under investigation.

The findings of this study implicate developmental exposure to dieldrin in mammary tumor promotion. The trans-placental and lactational transfer of dieldrin to offspring and bioaccumulation of this persistent pesticide in mammary tissues is cause for concern given that developing mammary structures are bathed in this toxicant with diverse biological actions. This study supports the epidemiological evidence for increased breast cancer and associated mortality in the human population with elevated dieldrin levels via tumor promotion. Furthermore, we provide evidence for altered neurotrophin/neurotrophin receptor expression in mammary tumors with elevated BDNF/TrkB in mammary tissues and tumors. The findings of this study support the roles of both dieldrin and TrkB signaling in mammary carcinoma and elucidation of the mechanism by which these factors influence breast cancer pathogenesis will be important for understanding toxicant-modulated carcinogenesis as well as for developing novel therapeutic targets and preventative strategies.

## Materials and Methods

### Ethics Statement

All animal experiments were approved by the Animal Research Ethics Board at McMaster University, in accordance with the guidelines of the Canadian Council for Animal Care.

### Animals

Sexually mature female (n = 117) and male (n = 60) FVB/N-TgMMTV-neu mice were purchased from a commercial breeder (Charles River) and bred in house. Animals were pair housed in polycarbonate cages at 22±2°C and 50±10% relative humidity on a 12:12 h light:dark lighting schedule. To exclude or minimize other exogenous sources of estrogenic agents, mice were provided with certified phytoestrogen-free rodent diet (TD96155; Harlan Teklad) and provided tap water *ad libitum* throughout the experiment.

### Breeding & Dosing

Female mice (8–10 weeks old) were housed with a male breeder mouse and examined daily for evidence of a sperm plug. Once a sperm plug was found the male mouse was removed and females were housed individually and pregnancy rate in each treatment group recorded. Day 1 of gestation was defined as the day a sperm plug was found. Dams (n = 29–30 per group) were randomly assigned to one of four treatment groups and treated daily by gavage for five days 2 weeks prior to mating and then once weekly throughout lactation until weaning with vehicle (Corn Oil) or dieldrin 0.45, 2.25, and 4.5 µg/g BW. Dieldrin doses were selected based on our previous experiments demonstrating this dosing strategy achieves tissue residue levels (10–30 ng/g) representative of those measured in human tissues reflecting background exposure [49]. At weaning (3 weeks), dams and male pups were culled and female pups began weekly dosing by gavage through 9 weeks of age. At 22 weeks all pups (n = 271) were anaesthetized with isoflurane and exsanguinated by cardiac puncture. At birth, post natal day 7 and weaning, the number, sex and weight of pups was recorded. Weight of female pups from weaning to sacrifice was recorded weekly. At sacrifice liver and kidneys were excised and wet weight recorded. Throughout the experiment, dams and offspring were monitored visually for signs of general toxicity including loss of body weight, hunched posture, ruffled coat, lacrimation, porphyria and startle response.

### Mammary Tumor Data

A midline incision was made along the spine and the skin removed to preserve the mammary gland structure. Mammary tumors were counted and volume calculated based on maximum and minimum diameter of tumors measured by callipers. Thoracic tumors were excised and preserved in RNA later (Sigma Chemical Co. Mississauga, ON).

### Histology and Immunohistochemistry

At the time of sacrifice, inguinal mammary glands, ovaries, uterus, kidneys, and liver were removed, fixed in 4% formaldehyde solution, dehydrated, and paraffin embedded. Sections (5 µm) were stained with hematoxylin and eosin (H&E) for histological analysis or processed for immunohistochemistry. Tumors arising in each location was assessed by a blinded observer and confirmed by a certified pathologist. Immunohistochemistry was performed using the Vectastain Elite ABC Kit (Vector Laboratories, Burlingame, CA) following the supplied instructions. Mammary gland sections were rehydrated and subjected to antigen retrieval using a low pH citrate buffer at 37°C, quenched for endogenous peroxidase activity with 3% H_2_O_2_ in methanol for 10 min at RT, and incubated at 4°C overnight with a primary antibody against TrkB at 1∶400 (Santa Cruz Biothechnology, Santa Cruz, CA). Slides were counterstained with Mayer's hematoxylin and negative controls run using pre-immune serum and positive controls using brain sections.

### Western Blot

Thoracic mammary tumors were processed for protein extraction by immersion in ice-cold RIPA buffer containing protease inhibitors (protease inhibitor cocktail tablets, Roche Diagnostics, Manheim, Germany) for 10 mins, and sonicated on ice. Extracts were then clarified by centrifugation and the concentration of protein in the supernatant determined by a Bradford microplate assay (BioRad, Hercules, CA, USA). Samples (20 µg protein) in reducing loading buffer were boiled and electrophoresed through 8% SDS-PAGE gels (Pierce, Rockford, IL). Separated proteins were electroblotted onto PVDF membrane (BioRad, Hercules, CA) and blocked in 5% non-fat powdered milk/Tris-buffered saline/Tween 20 (TBST) for 1 h. Primary antibodies used were anti-TrkB, rabbit mAb 1∶200 (Cell Signaling, Danvers, MA); anti-BDNF, 1∶200 (Santa Cruz Biotechnology Inc., Santa Cruz, CA) and anti-GAPDH, 1∶5000 (Abcam, Cambridge, MA). Blots were incubated in primary antibody overnight at 4°C then washed and incubated in secondary antibody-HRP conjugates for 1 h at RT (goat anti-rabbit at 1∶5000; Amersham Biosciences, Pittsburgh, PA). Blots were then washed extensively and immunoreactive proteins were visualized by using enhanced chemiluminescence (Amersham Pharmacia Biotech, Oakville, ON) and by exposing the membrane to Amersham Hyperfilm ECL (GE Healthcare Ltd. Buckinghamshire, UK). The optical density of the immunoreactive bands for TrkB, BDNF and GAPDH were quantified using ImageJ software. Density for TrkB and BDNF was corrected for background and normalized to the density of the corresponding band for GAPDH. The data were expressed as a ratio of the optical densities of TrkB or BDNF to GAPDH (whose expression was unaffected by treatment).

### Quantitative Real Time PCR

Total RNA was isolated from mammary tumors (preserved in RNAlater, Sigma Aldrich, St. Louis MO) using a Qiagen RNeasy mini kit with on-column DNAse digestion (Qiagen, Mississauga, ON) as per manufacturer instructions. Following confirmation of RNA integrity by gel electrophoresis and quantification by spectrophotometry, cDNA was reverse transcribed using random primers (Invitrogen, Burlington, ON). Real time PCR was performed with murine gene specific primers using an Applied Biosystems 7900HT Fast Real Time PCR System (Applied Biosystems, Foster City, CA). Gene- and species-specific primers were designed for neurotrophins NGF, BDNF, NT-4/5 and neurotrophin receptors TrkA, TrkB, TrkC, p75NTR using OligoPerfect™ (Invitrogen, Burlington ON) and ePCR software (http://www.ncbi.nlm.nih.gov/sutils/e-pcr/reverse.cgi). Table S2 shows the sequence of the forward and reverse primers, amplicon lengths, and accession numbers of the genes analyzed. Control reactions without cDNA and a no RT control were run to verify the absence of primer dimerization and genomic DNA contamination respectively. PCR amplification was carried out in a 20 µl reaction volume containing 5 ng of cDNA, 1.8 µM each of forward and reverse primers, and 10 µl Fast SYBR Green Master Mix (Applied Biosystems). The PCR reactions were initiated with denaturation at 95°C for 10 min; followed by 50 amplification cycles at 95°C for 15 s and 60°C for 1 min. Following amplification, to confirm the presence of a single amplification product, PCR products were subjected to a dissociation stage and derivative curve analysis as well as product separation on a 4% agarose gel stained with ethidium bromide. Samples were run in triplicate and results were averaged. CT was calculated by anaysis software SDS 2.2.1 (Applied Biosystems). Analysis of gene expression changes were calculated according to the method of Livak *et al.* using the 2^−ΔΔ*C*T^ method [50]. The normalized expression ratio (fold induction) was calculated by 2^−ΔΔ*C*T^ = fold induction. Statistical analyses were performed using the ΔC_T_±SD values.

### Statistical Analysis

Statistical analysis was conducted using SigmaStat (SPSS Inc., Chicago, IL.). All data are reported as the mean±standard error of the mean (SEM) unless otherwise noted. Treatment effects were determined by one way analysis of variance (ANOVA) and significant difference between the groups was determined by pairwise multiple comparisons using Dunn's test. Where the normality test failed, data were compared by ANOVA on ranks. The accepted level of statistical significance was set at *p*≤0.05.

## Supporting Information

Table S1 Breeding Data. Dams were treated daily for 5 days, 2 weeks prior to mating and then weekly dosing through weaning with vehicle of 0.45, 2.25, and 4.5 µg dieldrin/g by gavage. Data are the mean (±SD) and differences between treatment groups were determined by one way ANOVA and appropriate post hoc comparison test. *p<0.05 versus control.
(0.04 MB DOC)

Table S2 PCR primers
(0.04 MB DOC)
