# Supplementary material for: Developmental and Lactational Exposure to Dieldrin Alters Mammary Tumorigenesis in Her2/neu Transgenic Mice
Source: PLoS One. 2009 Jan 28;4(1):e4303. doi: 10.1371/journal.pone.0004303 (PMC2628733; doi:10.1371/journal.pone.0004303)
Supplement: Table S1 — Breeding Data. Dams were treated daily for 5 days, 2 weeks prior to mating and then weekly dosing through weaning with vehicle of 0.45, 2.25, and 4.5 µg dieldrin/g by gavage. Data are the mean (±SD) and differences between treatment groups were determined by one way ANOVA and appropriate post hoc comparison test. *p<0.05 versus control. (0.04 MB DOC) [file pone.0004303.s001.doc]

**Supplementary Information Table S1.** Reproductive performance of Dams.

|  |  | **Dieldrin** | | |
| --- | --- | --- | --- | --- |
|  | **Vehicle** | **0.45 μg/g** | **2.25 μg/g** | **4.5 μg/g** |
| No Dams | 30 | 29 | 29 | 29 |
| No. Dams pregnant | 28 | 26 | 25 | 12 * |
| Mean No. of live pups at birth | 7.3 ± 3.1 | 7.3 ± 2.0 | 6.9 ± 2.5 | 6.9 ± 2.6 |
| Mean No. Female pups (Day 7) | 3.1 ± 2.2 | 3.0 ± 1.3 | 3.5 ± 1.4 | 2.6 ± 1.3 |
| Mean No. Male pups (Day 7) | 3.1 ± 1.5 | 3.7 ± 1.7 | 3.0 ± 1.5 | 2.4 ± 1.4 |
| Mean No. Female pups Weaned | 3.1 ± 2.2 | 3.1 ± 1.5 | 3.4 ± 2.0 | 2.6 ± 1.3 |
| Mean No. Male pups weaned | 3.0 ± 1.5 | 3.7 ± 1.7 | 2.9 ± 1.6 | 2.4 ± 1.4 |
| Total No. Female pups Weaned | 85 | 81 | 85 | 20 |

Dams were treated daily for 5 days, 2 weeks prior to mating and then weekly dosing through weaning with vehicle of 0.45, 2.25, and 4.5 μg dieldrin/g by gavage. Data are the mean (± SD) and differences between treatment groups were determined by one way ANOVA and appropriate post hoc comparison test. *p<0.05 versus control.
