# Supplementary material for: Developmental and Lactational Exposure to Dieldrin Alters Mammary Tumorigenesis in Her2/neu Transgenic Mice
Source: PLoS One. 2009 Jan 28;4(1):e4303. doi: 10.1371/journal.pone.0004303 (PMC2628733; doi:10.1371/journal.pone.0004303)
Supplement: Table S2 — PCR primers (0.04 MB DOC) [file pone.0004303.s002.doc]

**Supplementary Information Table S2.** Real Time PCR primers

| Accession no. | Gene | Length (bp) | Forward primer | Reverse primer |
| --- | --- | --- | --- | --- |
| M35075.1 | NGF | 159 | **GCAGTGAGGTGCATAGCGTA** | **CTGTGTCAAGGGAATGCTGA** |
| NM_007540.4 | BDNF | 140 | **CAGTGACAGGCGTTGAGAAA** | **AACGCCCTCATTCTGAGAGA** |
| NM_001033124.1 | TrkA | 75 | CGTCATGGCTGCTTTTATGG | ACTGGCGAGAAGGAGACAG |
| X17647 | TrkB | 192 | TGGTGCATTCCATTCACTGT | CTTGGCCATCAGGGTGTAGT |
| BC139764.1 | TrkC | 123 | **GCCAGAGCCTTTACTGCATC** | **CTCCTTCTCGGACAGTCAGG** |
| AF105292 | P75NTR | 145 | **CTGCTGCTTCTAGGGGTGTC** | **GTTCACACACGGTCTGGTTG** |
| NM_008084 | GAPDH | 101 | CATCAAGAAGGTGGTGAAGC | GGGAGTTGCTGTTGAAGTCG |
